# Long-Term Care Policy and Practice

**DOI:** 10.1093/geroni/igaf122.1011

**Published:** 2025-12-31

## Abstract

Long-term care is a necessity for over 1 million older adults. This session will address both resident care issues and policy opportunities.

